# Toronto Aortic Stenosis Quality of Life Questionnaire (TASQ): Validation in Polish Patients with Aortic Stenosis

**DOI:** 10.3390/jcm14072502

**Published:** 2025-04-07

**Authors:** Natalia Świątoniowska-Lonc, Krzysztof Ściborski, Rima Styra, Claudia M. Lüske, Kinga Węgrzynowska-Teodorczyk, Derk Frank, Peter Bramlage, Waldemar Banasiak, Adrian Doroszko

**Affiliations:** 1 Department of Cardiology, Centre for Heart Diseases, 4th Military Hospital, 50-981 Wroclaw, Poland; k.sciborski@op.pl (K.Ś.); kinga.wegrzynowska@awf.wroc.pl (K.W.-T.); banasiak@4wsk.pl (W.B.); adrian.doroszko@gmail.com (A.D.); 2 Center for Mental Health, University Health Network, Toronto, ON M5G 2M9, Canada; rima.styra@uhn.ca; 3 Institute for Pharmacology and Preventive Medicine, 49661 Cloppenburg, Germany; claudia.lueske@ippmed.de (C.M.L.); peter.bramlage@ippmed.de (P.B.); 4 Faculty of Physiotherapy, University of Health and Sport Sciences in Wroclaw, 50-375 Wroclaw, Poland; 5 University Clinical Center Schleswig-Holstein, 24105 Kiel, Germany; derk.frank@uksh.de; 6 Clinical Department of Cardiology, Faculty of Medicine, Wroclaw University of Science and Technology, 51-612 Wroclaw, Poland

**Keywords:** aortic stenosis, cardiovascular disease, quality of life, quality of life measures

## Abstract

**Background/Objectives:** Quality of life (QoL) is recognized as a clinically significant outcome measure among patients with aortic stenosis (AS). However, there is no validated, AS-specific questionnaire available in Poland for assessing the QoL in AS patients. The aim of the study was to determine the psychometric properties of the Polish version of the Toronto Aortic Stenosis Quality of Life Questionnaire (the TASQ). **Methods:** The study involved 113 patients with severe AS (including 59 women), aged 74 to 82 years [mean age 77 years], hospitalized at the department of cardiology in 2024. The standardized questionnaires were used to assess the level of QoL, the TASQ, and the Minnesota Living with Heart Failure Questionnaire (the MLHFQ). **Results:** The mean QoL level assessed by the TASQ was 60.72 ± 22.82. The Cronbach’s alpha for the entire TASQ was 0.919, for the emotional impact subscale 0.873, and for the physical limitation subscale 0.861. Satisfactory values of fit measures were obtained for a five-factor structure (RMSEA < 0.01; CFI > 0.99). The loadings of each item were statistically significant (*p* < 0.001). The MLHFQ score correlated significantly (*p* < 0.001) and positively (r > 0) with the score on the scales of physical symptoms (r = 0.479), physical limitations (r = 0.662), social limitations (r = 0.597), emotional impact (r = 0.638), and overall QoL (r = 0.712). **Conclusions:** Patients with severe AS exhibit low QoL. The TASQ has very good psychometric properties and can be used to assess the QoL in the population of Polish patients with AS.

## 1. Introduction

Aortic stenosis is a common valvular disorder that leads to increased left ventricular afterload [1,2]. The epidemiology of aortic valve disease varies, and it is estimated that approximately 2% to 9% of patients over 75 years old have severe AS [3]. This number is expected to double or triple in future decades due to the aging of the population [4].

Symptoms such as exertional dyspnea or fatigue develop gradually after a long asymptomatic latent period [5]. Patients may develop chest pain, heart failure (HF), and syncope [6]. Imaging modalities are crucial in the assessment and management of patients with aortic valve disease, especially asymptomatic patients [7]. The primary technique is transthoracic echocardiography (TTE), which assesses valve morphology, the extent of valve damage, and the impact on left ventricular function and pulmonary circulation [8]. Exercise stress echocardiography is indicated in asymptomatic AS patients to unmask symptoms, to measure the degree of exercise-induced increase in mean transaortic pressure gradient, and to assess pulmonary hemodynamics [9,10]. For more accurate risk stratification, modern three-dimensional imaging techniques such as 3D echocardiography, MDCT, and CMR are used [8]. In more difficult cases, such as low-flow AS, echocardiography with dobutamine and MDCT assessment of calcification are helpful [1]. PET and CMR are also playing an increasingly important role in predicting disease progression, but require further study before implementation into routine clinical practice [7].

The treatment of AS includes surgical aortic valve replacement (SAVR) or transcatheter aortic valve implantation (TAVI) [8]. The choice of treatment should be based on the comprehensive Heart Team assessment based on analysis of the patient’s clinical status, procedural feasibility, team experience, anticipated benefits, and a personalized assessment of operative risk and chances for improving QoL [8,11].

QoL is a multidimensional concept encompassing physical, mental, and social aspects, as well as the patient’s ability to perform daily activities, including occupational functioning [12]. Researchers recognize QoL as a clinically significant outcome measure among patients with AS [13,14,15,16,17].

The treatment of AS not only reduces the risk of major cardiovascular and cerebrovascular events but also alleviates symptoms such as dyspnea, chest pain, and fatigue, which directly translates into improved QoL [18,19]. QoL assessment is a key tool in the analysis of therapeutic outcomes from the patient’s perspective, especially in a population with limited life expectancy [13,20]. Traditional indicators, such as mortality and morbidity, do not provide complete information about the physical, emotional, and psychological state of patients but can be supplemented with a subjective assessment of the healing process after treatment [4,21]. In addition, the identification of key risk factors and predictors of QoL could help the Heart Team to more accurately inform patients about the expected individual benefits of the procedure. Although it has already been shown that patients experience improvements in symptom reduction after valve replacement, there is still a lack of large-scale studies on the impact of treatment on physical well-being, psychological well-being, and QoL [22].

The available generic measures of QoL, such as the SF-12 [23] or EQ-5D [24], assess patients’ general health and well-being but do not take into account the specific symptoms and functional limitations resulting from aortic stenosis. This disease is associated with characteristic symptoms such as dyspnea, chest pain, or reduced exercise tolerance, which may not be sufficiently addressed by generic scales. Therefore, it is necessary to use a more specialized tool, such as the TASQ, which better reflects the impact of the disease on patients’ daily functioning and allows a more accurate assessment of treatment effects. The only validated disease-specific questionnaire used to assess QoL in patients with AS is the TASQ, which has excellent psychometric properties [25]. Several language versions of the TASQ have been developed, including English [25], French, German, Italian, and Spanish [26]. The TASQ has also been evaluated for convergent theoretical validity by comparing it with the generic Short Form-12 version 2 (SF-12v2) [26] and the heart-failure-specific Kansas City Cardiomyopathy Questionnaire (the KCCQ) [25,26] obtaining significant correlations between the KCCQ and SF-12v2 in the domains related to physical symptoms, physical limitations, emotional impact, and social limitations. In the past, the KCCQ scale was widely used in studies on AS, as there are some similarities between symptoms of severe AS and symptoms of HF unrelated to valvular disease [26,27,28]. However, the KCCQ focuses on symptoms characteristic of HF and does not account for parameters specific to AS [26]. Furthermore, the treatment of severe AS and its potential outcomes differ from therapies for other types of HF and cannot form the basis for assessing QoL in AS patients [25,26].

Currently, there is no validated, AS-specific questionnaire available in Poland for assessing QoL in AS patients. Available Polish studies have used generic questionnaires to assess QoL [13,15,16,29]. Generic questionnaires may not capture specific symptoms and health concerns related to particular conditions, such as AS. Additionally, general questionnaires may not reflect the impact of specific diseases on QoL. Therefore, the aim of this study was to determine the psychometric properties of the Polish version of the TASQ.

## 2. Material and Methods

### 2.1. Ethical Considerations

The study was approved by the Bioethics Committee of the Lower Silesian Medical Chamber in Wroclaw, Poland (approval no. 02/BOBD/2024, approval date on 10 January 2024). All participants provided written informed consent after a thorough explanation of the study procedures. The study was conducted in accordance with the principles of the Declaration of Helsinki.

### 2.2. Study Design and Settings

The current research has a cross-sectional, observational design. The study was conducted from January 2024 to September 2024. A total of 130 patients with severe AS, diagnosed according to European Society of Cardiology criteria, were initially qualified for the study. Among them, 10 declined to participate. In the first stage of qualification, 120 patients meeting the criteria were included. However, seven patients withdrew their consent. Ultimately, 113 patients aged 74 to 82 years (mean age 77) participated in the study. These patients were referred to the TAVI coordinator after qualification to TAVI by the Heart Team. The study qualification was carried out by a trained team consisting of the TAVI coordinator and an interventional cardiologist.

### 2.3. Inclusion Criteria

Inclusion criteria were written informed consent to participate in the study, age over 18 years, diagnosis of severe AS, and cognitive status, allowing understanding of the study’s purpose and methods, as well as completion of the questionnaire (MMSE: score ≥ 18).

### 2.4. Study Procedures

All involved patients assessed their QoL using the TASQ after the Heart Team qualification. After signing an informed written consent to participate in the study, all study participants were evaluated for cognitive impairment (as study exclusion criteria) using the 11-question-based Mini-Mental State Examination (the MMSE). Patients who scored ≥ 18 points on the MMSE received a paper-based TASQ questionnaire from the TAVI coordinator and completed it independently. After completing the questionnaire, they returned it to the TAVI coordinator, who then entered the patient’s responses into a spreadsheet along with socio-clinical information, which is presented in Table 1. The TAVI coordinator was responsible for preparing an anonymized version of the data, which was then passed to the study co-authors for the preparation of the results.

### 2.5. Research Tools

Two standardized questionnaires were used in the study: the TASQ [25] and the MLHFQ [30].

The TASQ is designed to assess QoL in patients with AS [25]. The TASQ considers specific disease symptoms, emotional state, daily functioning, and overall life satisfaction. It consists of 16 questions grouped into 5 domains: physical symptoms (Questions 1 and 14), physical limitations (Questions 3, 6, 7, and 15), social limitations (Questions 4 and 5), emotional impact (Questions 2 and 8 to 13), and health expectations (Question 16). Each question has a maximum score of 7, which reflects the severity of symptoms or the accuracy of the statement (1—not very much, 7—very much), giving the complete questionnaire a maximum total score of 112, with a higher score indicating improved QoL. The scores for each domain are summed to create an overall score for each subscale, as well as an overall TASQ score. Higher scores on the TASQ indicate better QoL, lower symptom severity, and a reduced impact of the disease on daily functioning. Permission to use the questionnaire and conduct a psychometric assessment in a Polish group of AS patients was obtained from the Institute for Pharmacology and Preventive Medicine. The study used the Polish version of the 27 February 2023 questionnaire provided by the Institute for Pharmacology and Preventive Medicine.

The MLHFQ is used to assess the QoL of patients with HF, considering the impact of the disease on their physical, emotional, and social functioning [30]. The MLHFQ consists of 21 questions addressing specific aspects of life for patients with HF, divided into two main domains: physical and emotional. The physical domain includes questions on the impact of HF on daily physical activities such as walking, climbing stairs, and self-care, as well as physical symptoms like fatigue, edema, and shortness of breath. The emotional domain assesses the intensity of frustration, depression, anxiety, and other emotions related to limitations caused by HF. In addition to the main domains, the MLHFQ also includes general questions about overall QoL and the global impact of the disease on the patient’s daily life. Each question in the MLHFQ is rated on a Likert scale from 0 (no impact) to 5 (maximum impact). The total score for the questionnaire ranges from 0 to 105 points. Higher scores indicate a lower level of QoL. The Polish version of the questionnaire was used in the study [31]. In our study, the MLHFQ was used to assess theoretical (convergent) accuracy.

The Mini-Mental State Examination (MMSE) is a tool used to assess cognitive function [32]. It assesses several areas of cognitive functioning, such as orientation (time, place), memory (repeating and recalling words), attention and counting (e.g., subtraction in memory), memory (recalling previously given words), language functions (e.g., naming objects, repeating sentences), and visual–spatial functions (e.g., drawing a geometric figure). The test consists of 30 questions, and a maximum score of 30 points can be obtained. The lower the score, the greater the cognitive impairment. The Polish version of the questionnaire was used in the study [33].

### 2.6. Preliminary Testing

For preliminary testing of the questionnaire, 30 cardiology department patients were recruited for the pilot study. After completing the translated questionnaire, respondents were asked (verbally by the interviewer or via an open-ended question) to explain what they believed each question and corresponding response meant.

### 2.7. Statistical Analysis

A comparison of quantitative variables in the two groups was performed using the Mann–Whitney test. Univariate and multivariate analysis of the effect of potential predictors on the quantitative variable was performed using linear regression. The results were rearranged as regression parameters along with 95 percent confidence intervals. The internal consistency of the tool was assessed using Confirmatory Factor Analysis (CFA). Reliability of the scales was assessed using Cronbach’s alpha. Normality of distribution was checked using the Shapiro–Wilk test. Theoretical accuracy (construct validity) was checked by calculating correlations between the TASQ and MLHFQ scores. These correlations were analyzed using Spearman’s correlation coefficient. The analysis assumed a significance level of 0.05, and all *p*-values below 0.05 were interpreted as indicating significant correlations. The analysis was performed in R software, version 4.4.2 [34], with the lavaan package [35].

## 3. Results

### 3.1. Patients’ Characteristics

The study included 113 patients with severe AS at a mean age of 77 [74–82] years (Table 1). Most respondents were female (52.21%), single (51.32%), and had completed secondary education (47.79%). The mean (SD) left ventricle ejection fraction (the LVEF) was 57.54 (10.8). Most patients were in New York Heart Association (NYHA) functional classes II (54.86%). The mean (SD) EuroSCORE II was 3.71 (3.51), the Charlson Comorbidity Index 5.2 (1.62), and the MLHFQ 37.62 (25.58).

### 3.2. Scale Characteristics and Internal Consistency

The level of QoL assessed by the TASQ is presented in Table 2. The Cronbach’s alpha for the entire TASQ questionnaire was 0.919; for the emotional impact subscale, it was 0.873; and for the physical limitations’ subscale, it was 0.861. For scales consisting of fewer than three items, Cronbach’s alpha was not calculated.

### 3.3. Internal Consistency—Confirmatory Factor Analysis

Since TASQ items are expressed on an ordinal rather than on a continuous scale, the Diagonally Weighted Least Squares (DWLS) method was used. The original TASQ structure is a five-factor one (Table 3).

Satisfactory values of fit measures were obtained for this structure (RMSEA < 0.06, CFI and TLI > 0.95, and SRMR < 0.08) [36] (Table 4).

The loadings of each item were statistically significant (Table 5). A reliability analysis of the questionnaire showed very good psychometric properties in the questionnaire. All items have positive discriminatory power (item-total correlation) and correlate positively with the other items included in the scale.

### 3.4. Theoretical (Convergent) Accuracy—Analysis of Correlations Between the TASQ and the MLHFQ

The MLHFQ score correlates significantly (*p* ˂ 0.05) and positively (r > 0) with the score on the scales of physical symptoms, physical limitations, social limitations, emotional impact, and overall QoL (Table 6). Thus, the higher the MLHFQ score, the higher the domains and the overall TASQ score.

## 4. Discussion

The TASQ is increasingly used to assess the QoL in patients with AS [25,26,37]. In this study, an attempt was made to evaluate the psychometric properties of the Polish version of the TASQ. To the best of our knowledge, this is the first study assessing the QoL of Polish patients with AS using the TASQ. Our study confirmed that patients with severe AS have a poor quality of life. Furthermore, the TASQ was found to have very good psychometric properties and can be used to assess QoL in the Polish AS patient population.

Psychometric analysis was conducted by evaluating Cronbach’s alpha coefficient, which measures the internal consistency of the research tool. According to the literature data, the optimal Cronbach’s alpha value should be ≥0.90 [38]. In our study, the Cronbach’s alpha for the entire TASQ was 0.919. A similar Cronbach’s alpha of 0.92 was obtained for the original version of the TASQ [25]. The reliability of the tool in the English, French, German, Italian, and Spanish versions was also confirmed in a cultural adaptation conducted by Frank et al. [26]. The Cronbach’s alpha was lower than that of the original version and our study results, at 0.891. Lower values may be associated with a disproportionate sample selection and the lack of a reliability assessment for each language version separately.

Since the TASQ has not yet been used among the Polish patient population, in addition to reliability analysis, convergent construct validity was assessed. To evaluate external validity, the specific MLHFQ was used, which has previously been employed to assess QoL in patients with AS [39,40]. The analysis revealed a significant positive correlation between the TASQ and MLHFQ scores, indicating that the TASQ is externally valid. The only TASQ domain that the MLHFQ did not correlate with was the health expectations domain. This indicates some shortcomings of the MLHFQ questionnaire in assessing QoL in a group of AS patients. Other studies have shown a positive correlation between the TASQ and the KCCQ [25,26] and demonstrated that the KCCQ is a valid and reliable tool for AS patients [41,42], although it does not assess specific variables related to AS symptoms and treatment.

In our study, in addition to evaluating the psychometric properties of the TASQ, we assessed the QoL level. The average overall QoL obtained by AS patients was 60.72 ± 22.82. A similar result was obtained in the study of the original version of the TASQ [25]. In contrast, in the study by Frank et al. [26], the overall QoL measured by the TASQ was higher, at 71.09 ± 19.43. It should be noted that the QoL measured by the TASQ may be influenced by differences in healthcare systems between countries, including socioeconomic conditions [43]. Studies show that AS patients with lower socioeconomic status (SES) often have a poorer prognosis and higher mortality rate [44]. This is due to several factors, including limited access to early diagnosis, delayed treatment, and lack of adequate support in the post-operative period. In addition, patients with lower SES may experience greater stress and financial burden, which negatively affects their mental health [29,45]. These problems can compound the perceived discomfort of disease symptoms such as shortness of breath, fatigue, and chest pain [13,15]. Compared with other validation studies [25,26], in our study, participants had the lowest QoL scores in the social and emotional domains. This may be related to the high percentage of single individuals in the study group and the low socioeconomic status of participants.

### Study Limitations

The study has several limitations. One of these is the small sample size and the single-center design of the study. Extending the validation to a larger, more diverse cohort across multiple centers would strengthen the generalizability of the results. The MLHFQ did not show a significant correlation with the health expectations domain of the TASQ, so further clarification or the use of additional validation instruments may be beneficial. Future studies should assess the external validity of the TASQ by including other validated measures of AS-related QoL, such as the Kansas City Cardiomyopathy Questionnaire (KCCQ), to further confirm the results. The study only assessed patients prior to TAVI. In addition, the psychometric properties of the TASQ were evaluated only in a group of patients qualified for TAVI. To further validate the stability of the questionnaire over time, it would be useful to assess test–retest reliability. Future studies should also assess how TASQ scores change after TAVI. It would be advisable to consider expanding the study to assess QoL after valve replacement procedures, both TAVI and SAVR.

## 5. Conclusions

Patients with severe AS exhibit a low QoL. The TASQ has very good psychometric properties and can be used to assess QoL in the population of Polish patients with AS.

## Figures and Tables

**Table 1:** Sociodemographic and clinical characteristics of the study group.

Parameter		Total (N = 113)
**Sex**	Male	54 (47.79%)
	Female	59 (52.21%)
**Age [years]**	Mean (SD)	77.26 (6.9)
	Median (quartiles)	77 (74–82)
	Range	41–91
**Marital status**	In a relationship/married	55 (48.67%)
	Unmarried	9 (7.96%)
	Separated/divorced	3 (2.65%)
	Widowed	46 (40.71%)
**Education**	None or primary	29 (25.66%)
	Secondary	54 (47.79%)
	Higher	30 (26.55%)
**BMI [kg/m^2^]**	Mean (SD)	28.91 (4.96)
	Median (quartiles)	28.01 (25.32–31.14)
**LVEF [%]**	Mean (SD)	57.54 (10.8)
	Median (quartiles)	60 (55–65)
	Range	25–75
**NYHA**	I	7 (6.19%)
	II	62 (54.86%)
	III	41 (36.28%)
	IV	3 (2.65%)
**CCS**	1	15 (13.27%)
	2	24 (21.24%)
	3	10 (8.85%)
	4	2 (1.77%)
**EuroSCORE II**	Mean (SD)	3.71 (3.51)
	Median (quartiles)	2.54 (1.8–4.26)
**Charlson Comorbidity Index**	Mean (SD)	5.2 (1.62)
	Median (quartiles)	5 (4–6)
**Minnesota Living with Heart Failure Questionnaire**	Mean (SD)	37.62 (25.58)
	Median (quartiles)	30.5 (16–57.75)

Abbreviations: SD: standard deviation; CCS: Canadian Cardiovascular Society classification; NYHA: New York Heart Association classification; LVEF: left ventricle ejection fraction.

**Table 2:** The level of quality of life measured by the TASQ.

TASQ	Mean	SD	Median	Min	Max	Q1	Q3
Physical symptoms	8.43	2.73	9	3	14	6	11
Physical limitations	17.93	6.94	19	6	28	12	25
Emotional impact	22.42	12.18	21	7	49	12	34
Health expectations	6.15	1.46	7	1	7	5.75	7
Social limitations	6.17	4.52	4	2	14	2	10
Total score	60.72	22.82	59	25	110	40.75	78

Abbreviations: SD: standard deviation; Q1: lower quartile; Q3: upper quartile; TASQ: Toronto Aortic Stenosis Quality of Life Questionnaire.

**Table 3:** TASQ structure.

Scale	Items
Physical symptoms	1, 14
Physical limitations	3, 6, 7, 15
Emotional impact	2, 8, 9, 10, 11, 12, 13
Health expectations	16
Social limitations	4, 5

**Table 4:** Fit indices of the tested TASQ.

Chi-Squared Test			RMSEA	CFI	TLI	SRMR
χ^2^	df	*p*				
44.525	100	<0.999	<0.01	>0.99	>0.99	0.054

Abbreviations: χ^2^: Chi-square test; CFI: comparative fit index; df: degree of freedom; RMSEA: root mean square error of approximation; TLI: Tucker–Lewis Index; SRMR: standardized root mean square residual.

**Table 5:** Loadings.

Scale	Item	Loading	*p*
Physical symptoms	1	0.710	*p* < 0.001 *
	14	0.728	*p* < 0.001 *
Physical limitations	3	0.908	*p* < 0.001 *
	6	0.834	*p* < 0.001 *
	7	0.816	*p* < 0.001 *
	15	0.550	*p* < 0.001 *
Emotional impact	2	0.690	*p* < 0.001 *
	8	0.558	*p* < 0.001 *
	9	0.889	*p* < 0.001 *
	10	0.727	*p* < 0.001 *
	11	0.468	*p* < 0.001 *
	12	0.646	*p* < 0.001 *
	13	0.820	*p* < 0.001 *
Health expectations	16	---	---
Social limitations	4	0.918	*p* < 0.001 *
	5	0.944	*p* < 0.001 *

* statistically significant (*p* < 0.05).

**Table 6:** Convergent construct validity.

TASQ	MLHFQ
	Spearman’s Correlation Coefficient
Physical symptoms	r = 0.479, *p* < 0.001 *
Physical limitations	r = 0.662, *p* < 0.001 *
Emotional impact	r = 0.638, *p* < 0.001 *
Health expectations	r = −0.198, *p* = 0.05
Social limitations	r = 0.597, *p* < 0.001 *
Total TASQ score	r = 0.712, *p* < 0.001 *

* statistically significant (*p* < 0.05). Abbreviations: r: Spearman’s correlation coefficient; TASQ: Toronto Aortic Stenosis Quality of Life Questionnaire; MLHFQ: Minnesota Living with Heart Failure Questionnaire.

## Data Availability

The original contributions presented in this study are included in the article. Further inquiries can be directed at the corresponding author.
